# THE EFFECTS OF POSTRETIREMENT WORK ON WELL-BEING OF RETIRED ADULTS

**DOI:** 10.1093/geroni/igad104.1062

**Published:** 2023-12-21

**Authors:** Edwin Chung, Gloria Lin, Dannii Yeung

**Affiliations:** City University of Hong Kong, Hong Kong, Hong Kong; City University of Hong Kong, Hong Kong, Hong Kong; City University of Hong Kong, Hong Kong, Hong Kong

## Abstract

There has been evidence that a growing number of retirees are reentering the labor force (DeSilver, 2016; Greenwald et al., 2017), possibly due to financial needs and seeking continuity in their preretirement job (Burkert & Hockfellner, 2017). However, the effects of post-retirement work on retirees’ well-being remain under-examined. This cross-sectional study thereby aims to examine the effects of post-retirement work in a sample of 768 younger and 530 older Hong Kong Chinese retirees (Mage = 65.1, SD = 2.72, range = 60 – 69, and Mage = 75.2, SD = 4.50, range = 70 – 95, respectively). Participation in post-retirement work, life satisfaction, cognitive functioning and hand-grip strength were measured. The results of two-way between-subject robust ANOVAs demonstrated that retirees engaging in post-retirement work exhibited lower life satisfaction (Q = 5.82, p = .02) but higher cognitive functioning (Q = 5.29, p = .02) than those without post-retirement work. Furthermore, as compared with younger retirees, older retirees engaging in post-retirement work exhibited higher cognitive functioning (Q = 3.66, p = .05) and stronger hand-grip strength (Q = 4.21, p = .04). These results remain significant even after controlling for sex, education, socioeconomic status, perceived health, and financial control. The findings of this study thus reveal the beneficial effects of post-retirement work on cognitive and physical health, but not on life satisfaction. The negative effect of post-retirement work on subjective well-being will be discussed in relation to the unsatisfactory working environment or employee benefits received by older workers in Hong Kong.

